# Underwater drag reduction via self-healing and robust air plastrons stabilized by candle soot-based coatings

**DOI:** 10.3389/fchem.2026.1814633

**Published:** 2026-05-01

**Authors:** Muhammad Imran Jamil, Arshia Komal, Mehboob Hassan, Mazloom Shah, Waqar Ahmed, Hafiz Muhammad Ali, Numan Ahmed, Fazal Haq, Sahid Mehmood, Shahid Iqbal, Meznah M. Alanazi, Shaimaa A. M. Abdelmohsen

**Affiliations:** 1 Department of Chemistry, Faculty of Science, Grand Asian University Sialkot, Sialkot, Punjab, Pakistan; 2 Department of Chemistry, University of Narowal, Narowal, Punjab, Pakistan; 3 Department of Bionano Engineering, Hanyang University, Ansan, Republic of Korea; 4 Mechanical Engineering Department, King Fahd University of Petroleum and Minerals, Dhahran, Saudi Arabia; 5 Interdisciplinary Research Center for Sustainable Energy Systems (IRC-SES), King Fahd University of Petroleum and Minerals, Dhahran, Saudi Arabia; 6 Yangtze Delta Region Institute (Huzhou), University of Electronic Science and Technology of China, Huzhou, Zhejiang, China; 7 Institute of Chemical Sciences, Gomal University, Dera Ismail Khan, Pakistan; 8 Key Laboratory for Special Functional Aggregated Materials of Ministry of Education, School of Chemistry and Chemical Engineering, Shandong University, Jinan, Shandong, China; 9 Nottingham Ningbo China Beacons of Excellence Research and Innovation Institute, University of Nottingham Ningbo China, Ningbo, China; 10 Department of Physics, College of Science, Princess Nourah bint Abdulrahman University, Riyadh, Saudi Arabia

**Keywords:** Cassie-Baxter superhydrophobicity, drag reduction, durable, healable plastron, Taylor-Couette

## Abstract

In marine environments, the increased drag force on vehicles impedes their speed underwater, leading to high energy consumption and reduced vehicle efficiency. Superhydrophobic coatings have gained remarkable attention regarding drag reduction. Due to the collapse of the air plastron, the change from Cassie–Baxter to Wenzel state at high fluid impalement pressures, and various chemical conditions, these coatings lose their ability to reduce drag in cold marine settings. Candle soot nanoparticles are deposited into an elastic binder to create a strong, environmentally friendly porous superhydrophobic covering with healable plastron to solve these difficulties. The interconnected nano-pores of the soot particles retain the drag reduction performance by robustly maintaining the air plastron under water. The water droplets bouncing on elastic soot coating cause drag reduction by reducing their contact time. The soot-coated copper ball enfolds the gas cavity volume 14.83 times greater than that of the bare ball while impacting water. The near-zero drag coefficient values of the soot-coated copper sphere confirm its efficient drag reduction performance. In Taylor-Couette flow, the soot-coated rotor showed a 60% drag reduction as compared to the bare rotor. The rheological experiments showed a 15.26% reduction in the apparent viscosity of glycerol and a 13.86% shear stress reduction on soot coated surface at −10 °C. The soot-coated boat covered 31 cm more distance at high speed than the uncoated boat. The plastron of candle soot coating showed stability under lake water, pond waters, super-cold water, liquid nitrogen, acidic and basic media, artificial seawater, different organic solvents, and sand abrasion. Moreover, a soot-coated aluminium plate floated on the surface of water while bearing a load. The durability, passive plastron stability, and self-cleaning of candle soot superhydrophobic coating have the potential for efficient drag reduction and are suitable for large-scale applications even in arctic environments in order to improve the fuel efficiency and substantial energy savings.

## 1 Introduction

Drag is a force that reduces the motion of an object in a fluid, influenced by its velocity, shape, and fluid properties, and is characteristically described by the drag coefficient (Bixler and Bhushan, 2013). The maritime sector transports up to 95% of commodities globally (Ahmadzadehtalatapeh and Mousavi, 2015). For moving objects when submerged in water, friction force can be equivalent to around 80% of the total resistance, as on the boat’s surface it takes up to 50%, while in submarines this extent relies on 70% of the fluid stream (Gu et al., 2014). The main reason is biofouling, caused by the adhering of macro/microorganisms to the vehicle’s surface in water, already a global marine issue that costs billions of dollars annually. Biofouling corrodes the vehicle’s outer surface and makes it rougher, ultimately increases the hydrodynamic drag as the vehicle passes through the water (Feng et al., 2022). For water vehicles, hydrodynamic drag not only causes significant energy consumption but also reduces their speed (Saranadhi et al., 2016) and 85% of fuel energy is consumed to overcome water resistance. The distance and speed of the vehicle are increased by 3.57% for every 10% reduction in drag under specific power and energy conditions (Yunqing et al., 2017).

Different strategies have been used to decrease the drag forces underwater, such as the Leidenfrost phenomenon (Vakarelski et al., 2016), the usage of polymer additives, active gas-generating methods, the supercavitation effect, superhydrophobic and slippery liquid-infused porous coatings. The Leidenfrost effect is limited due to the consumption of continuous heat and possible explosions. Expensive cavitations are required for cavitation and supercavitation effect to maintain constant steam lubrication for reducing drag forces under water (Jia et al., 2022). Supercavitation is also limited to certain fast-moving vehicles. Active gas-generating techniques such as bubble injection have limited practical applicability because they need special equipment and extra energy to produce gas. Due to their inability to effectively decrease drag in harsh environments, these techniques are both costly and unsuitable for large-scale deployments (Liao et al., 2023). Frictional drag has been considerably lowered by 24% via repeated microbubble injection in turbulent boundaries (Tanaka et al., 2023). However, the instability of bubbles and proneness to cracks increase noise and drag force. The drag reduction is not achieved when the bubbles are extremely small. Moreover, its practical use is further hindered by its unusual high energy consumption and dependence on electricity. The Leidenfrost surfaces and supercavitation methods lead to the formation of partial slip boundary conditions but only possible at high temperatures and extreme flow velocity rates that restrict their use for real marine environment (Vakarelski et al., 2016).

Polymer additives are utilized to reduce drag even with a minor amount. 20.18% drag reduction is achieved by using polyethylene oxide (Chang et al., 2024). However, their rapid degradation under shear is the main reason behind their limited usage (Gu et al., 2020). Drag is also reduced by the dynamic lubrication provided by slippery liquid-infused surfaces (SLIPSs) (Solomon et al., 2014). A 16% drag reduction is achieved in the Couette flow by using silicone SLIPS made by PDMS and dimethyl silicone oils (Wu et al., 2024). These liquid-infused surfaces have the potential to reduce drag (Vega-Sánchez et al., 2022; Zhu et al., 2022), but the evaporation of entrapped lubricant, scalability, high cost of materials and equipments, multi-step fabrication methods, and lubricant depletion under shear stress limit their practical usage (Pakzad et al., 2022).

Therefore, to cope with the aforementioned problems, durable superhydrophobic coatings have drawn a lot of attention over time to competently reduce underwater drag (Golovin et al., 2016; Vakarelski et al., 2012). Natural creations like lotus leaves (Sarma et al., 2022), dolphin (Yu et al., 2020) and shark skin (Bixler and Bhushan, 2012), penguin feathers, and butterfly wings (S et al., 2021; Chen et al., 2024) with unique morphology can reduce drag due to distinct surface wettability and low energy (Xu et al., 2023). Thus, inspired by the hydrophobic phenomenon in nature, locating a fresh source of superhydrophobic materials with consistent wettability would be quite helpful for drag reduction (Kim et al., 2022).

Superhydrophobic coatings (water CA, 150°), are intended to increase drag reduction by trapping air layers from water (Park et al., 2021). The trapped layer of air is called a plastron (also known as air pockets, air cushion, air bubbles, or entrapped air layer) which is crucial for superhydrophobic Cassie–Baxter state. Superhydrophobic coatings are characterized by Cassie–Baxter and Wenzel state (Cassie and Baxter, 1944). In the Cassie–Baxter state, the micro- or nano-structures trap the air layer and promote the formation of a thin air plastron between the surface and the water, keeping both phases in critical contact (Papadopoulos et al., 2013). A new wetting state called the “Wenzel” state forms if the liquid-vapor interface penetrates the air gap between the structures; this depends on the fluid’s thermophysical characteristics, the environment, and the spatial arrangements of the structures.

For ensuring long-lasting stable marine operation and fluid transfer pipelines through high-speed underwater movement, attention is required to reduce drag. Superhydrophobic coatings are progressively used in a number of industries such as marine, wind power, desalination, aviation, and oil/water separation (Feng et al., 2022). In recent studies, a superhydrophobic coating was developed by combining perfluorodecyltriethoxysilane (FOTS) with TiO_2_ nanoparticles (Hwang et al., 2017) and fluorinated silica particles, which resulted in a 27% reduction in drags (Liu et al., 2019). In turbulent flow, 20% decreased drag was observed when hydrophobic silica nanoparticles were treated with HMDS (hexamethyl disulphide) and beeswax to make a superhydrophobic epoxy coating (Fakhri et al., 2023; Pakza et al., 2020). The naturally hydrophobic polytetrafluoroethylene was sanded to produce superhydrophobic surfaces which reduced drag by up to 27% (Song et al., 2014). Inspired by spiders and fire ants, a floatable superhydrophobic device was designed by using fabricated aluminium plates. Superhydrophobicity was induced on a nickel-aluminium-bronze surface with a contact angle of 163° by using a picosecond laser (Tang et al., 2022).

The sol-gel method was utilized to develop a superhydrophobic nano-coating on an aluminium disc, resulting in a drag reduction of up to 30% in laminar flow and 15% in turbulent flows as compared to a smooth uncoated aluminium disc (Moaven et al., 2013). Superhydrophobic coatings significantly reduce underwater drag by facilitating the formation of smooth air layers and spreading air bubbles, achieving a 20% reduction in drag (Vakarelski et al., 2016). Femtosecond laser technology was used to introduce a drag-reducing micro-channel on the silica glass surface that resulted in the formation of a firm superhydrophobic surface with a micro-pillar array (Xu et al., 2023). The superhydrophobic surface developed on the aluminium substrate using anodizing and etching, spin-coated with fluorinated polymer, demonstrated up to 15% drag reduction in water flow rate conditions (Barbier et al., 2012). By attributing superhydrophobicity with a porous surface, 76% frictional drag reduction was achieved (Sun et al., 2022). A coating consisting of hydrophobic copper particles and polydimethylsiloxane demonstrated a 26% reduction in drag (Cheng et al., 2015). Using the flame soot layer as a nanoimprint template through calcination, superhydrophobic TiO_2_ film coating was also fabricated (Liu et al., 2013). These superhydrophobic coatings reduce the drag force underwater. But besides all that, the complicated nanofabrication procedures reduced mechanical durability (Sutar et al., 2020; Zulfiqar et al., 2018), and high cost hinder the practical use of the coatings at large scale. Also, these superhydrophobic coatings lose their potential in saline environments with extremely basic or acidic conditions (Brennan et al., 2015; Qu et al., 2010). Furthermore, the superhydrophobic coatings with poorly stable air plastrons eventually damage the superhydrophobicity permanently, ultimately serving no advantage for drag reduction under water (Erbil, 2020; Liravi et al., 2020; Nilsson et al., 2010).

Here, a low-cost, robust candle-soot-based superhydrophobic coating using hydrophobic RTV-1 (Room Temperature Vulcanizing) as an effective binder is proposed, which is a promising way to handle persistent drag challenges effectively. The durable Cassie–Baxter superhydrophobicity and passive air plastron are introduced by depositing candle soot nanoparticles into a binder-coated aluminium substrate. The developed coating exhibits strong water-repellent behavior and generates air plastrons on its immersion in water. Soot-coated copper balls impacted in water with specific velocity surrounded by stable teardrop-shaped sphere-in-cavity with a volume of 9.26 and 14.83 times greater than the volume of bare copper balls with 10 mm and 20 mm diameter respectively. A Taylor-Couette rotor test is performed where torque signals are reduced by 54% and 60% proficiently for soot coating as compared to the uncoated surface on 60 rad/s and 240 rad/s rotation at 25 °C. In a rheological experiment, apparent viscosity as a function of the shear rate is reduced by 15.26% on the soot-coated surface at a 1,000 µm gap height. The reduction in shear stress as a function of temperature is 13.86%, revealing the low adhesion of the soot coating and better mechanical strength even at low temperatures (1 °C to −10 °C). Superhydrophobic candle soot coating demonstrates long term stability in super cold water, liquid nitrogen, lake and pond water, basic and acidic media, artificial seawater, and organic solvents. The soot-coated boat covered 31 cm more than the uncoated boat at high speed, which confirms drag reduction underwater. The developed soot coating exhibits 3.68 MPa tensile strength and remains stable even after sand abrasion test. This candle-soot-based superhydrophobic coating is durable, exhibiting healable plastron, high stability at near-zero temperature, anti-fouling, anti-corrosive, self-cleaning and load-bearing abilities. These properties make it a promising candidate for drag reduction, anti-fouling, anti-corrosive, and oil-water separation applications in maritime transportation and offshore stations, providing enhanced competency and substantial energy savings.

## 2 Materials and methods

### 2.1 Materials

Paraffin wax candles were purchased from the local market of Narowal, Pakistan. RTV-1 silicone (YW 9509 W) binder was purchased from Shen Zhen Xing YONGWEI Silicone Co., LTD. China. The substrates aluminium plates, microscopic glass slides, and silicon wafers were purchased from Urdu bazar Lahore, Pakistan. Ethanol, acetone and deionized water were purchased from the local market of Sialkot, Pakistan.

### 2.2 Fabrication of superhydrophobic coating

The substrates aluminum plates and glass slides were cleaned with acetone and ethanol respectively. RTV-1 was spin-coated for 60 s at 5,000 rpm to in order to get a smooth layer of the binder on the substrates. The binder-coated substrate was then moved back and forth to guarantee even soot nanoparticle deposition while being held over the candle flame (2.9 cm above the wick, as shown in Supplementary Figure S1). After 2 minutes of soot deposition, the RTV-1 coated glass slide was completely covered in soot particles and became black, as shown in Supplementary Figure S2. Then, the extra non-adhered soot nanoparticles were detached by using air pressure before the commencement of the drag reduction and mechanical durability assessments. The propane hand torch and butane gas flame is also used as an alternative heating source instead of candle flame for large scale applications.

### 2.3 Characterization of chemical composition, morphology and wettability of coating

We used an Alpha Bruker ATR spectrometer to capture Fourier-transform infrared (FTIR) spectra between 500 and 4,000 cm^-1^. The roughness of the developed coating was measured by operating AFM (Agilent 5,500) with tap mode and the morphology of the fabricated surface was measured by operating SEM (Hitachi SU-8010) with 3.0 kV energy of beam and 10.0 mm working distance. SINDIN Co. Ltd., China’s SDC-100 optical contact angle goniometer was used to measure the contact angle (CA) of water droplet (10 µL). The contact angle hysteresis (CAH) of water was measured by Data Physics OCA 20, California. For the same sample, measurements were taken from at least five diverse positions and then averaged to acquire reliable values.

### 2.4 Sphere-in-cavity configuration

In order to create free slip boundary conditions, the spherical copper balls (diameter, D_S_ of 10 mm and 20 mm) coated with candle soot nanoparticles, were used. These balls were immersed in water to observe the sphere-in-cavity. These soot-coated copper balls (mass of the sphere, m_S_ = 4.42 g and 35.14 g for a diameter of the sphere, D_S_ = 10 mm and 20 mm, respectively) were left to drop in a particularly designed fluid tank of 20 cm × 20 cm cross-section with a height of 2 m. Superhydrophobic copper balls descended from a height of 90 cm in the air to a water-filled deep tank to construct the sphere-in-cavity assembly for moving balls. Using high-speed and high-resolution video cameras with 2000 fps, the fall of bare copper balls and soot-coated copper balls were recorded simultaneously. Snapshots taken at high magnification from recorded videos were used to estimate the volume of the cavity.

### 2.5 Torque signal reduction on Taylor-Couette rotor

For the drag reduction experiment, two stainless steel cylindrical Taylor-Couette rotors were employed, sharing identical dimensions; the radius of the inner cylinder (R) was 20.5 mm, the radius of outer cylindrical cup (R′) 24 mm, and the height of the inner cylinder (H) 55 mm. Using a commercial rheometer, the Taylor-Couette flow was created. A stainless steel cylindrical cup was filled with water with a density (ρ) 9.97 × $10^{2}$ kg/m and dynamic viscosity (µ) 0.89 m Pas. One Couette rotor was coated with RTV-1 binder, followed by candle soot particle deposition (superhydrophobic) while the other in the bare state (uncoated rotor) was separately immersed in deionized water at room temperature (25 °C). High-accuracy torque measurements were made on the submerged custom-made rotor with a constant angular velocity at 60 rad/s and 240 rad/s using a torque transducer in a controlled stress rheometer. Both soot-coated and uncoated rotors underwent at least five trials to measure torque signals.

### 2.6 Rheological experiments

In order to apply a shear rate and evaluate the viscous torque of the sample, an AR-G2 rheometer (TA Instruments, ARES-G2) with a standard plate–plate geometry was used. A stainless-steel clamp with a radius of 8 mm was rotated at a specific angular velocity, powered by a given torque. The lower Peltier plate was coated with candle soot nanoparticles. The upper plate was brought down to establish a zero-gap height, making sure the parallelism. The mean gap height error caused by plate-plate configuration was calculated beforehand as narrow fluid gaps can cause alignment errors for conventional rotational rheometers. The test liquid (glycerol solution-70 wt.%) confined in the space between the clamp and the stationary SH coating surface was sheared by the clamp. The torque acting on the clamp and the shear rate differ from the non-slip condition if there is a slippage effect on the interface of the SH coating and the liquid. Measurements were taken at different gaps, ranging from h = 300 μm–1,000 μm, in order to reduce the effects of these errors by introducing the test liquid (glycerol solution-70 wt.%) on the test surface. The torque applied to the clamp was determined for a range of given shear rates between 10 ≤ γ ≤ 100 $s^{-1}$ , following the zero-gap calibration. The temperature of the test liquid was kept at 25 °C throughout all of the rheological evaluations.

### 2.7 Durability and air plastron regeneration

1 M acidic solution (pH 1.7) was prepared by adding 400 μL of 98% concentrated sulfuric acid in 400 mL of Millipore-Q water. To prepare the basic solution (pH 10), 5 g of sodium hydroxide (NaOH) pellets were dissolved in 500 mL Millipore-Q water. The artificial seawater mimicking the composition of natural seawater was prepared by using potassium bromide (0.101 g), sodium bicarbonate (0.201 g), potassium chloride (0.69 g), calcium chloride (1.16 g), sodium sulphate (4.09 g), magnesium chloride (5.20 g), and sodium chloride (24.53) in distilled water (L). Different organic solvents including tetrahydrofuran, benzene, toluene, butyl acetate, ethyl acetate, hexane, ethanol, methanol, and isopropyl alcohol were used to check the stability of the plastron after the evaporation of solvents. The superhydrophobic soot coating was immersed in deionized water with pH 7 for 6 months at room temperature. The stability of the soot coating was assessed at low temperatures by immersing it in super-cold water (−2 °C) and liquid nitrogen (−180 °C). As well, the coating’s resistance towards extreme chemical conditions were validated by its immersion in lake and pond water for 8 months, 7 days of immersion in highly basic and acidic medium, and 10 months in artificial seawater and organic solvents, separately within experimental containers. Additionally, the self-cleaning capability of the prepared coating was demonstrated by purposely spotting dust onto the soot coating positioned at a slope angle of ∼10° and subsequently rinsed with water. A digital high-resolution camera was used to capture the self-cleaning consequence at 60 frames per second.

### 2.8 Mechanical durability

To assess the mechanical durability of the passive plastron and superhydrophobic coating under water, a sand abrasion test was conducted. To abrade the soot coating, 200-g weight was used to press the coating against the sandpaper, later the coating moved along the ruler for 200 cm (Tesler et al., 2024). Moreover, the mechanical strength of the coating was also confirmed at low temperatures (1 °C to −10 °C) by performing a rheological shear-stress test. A silicon rubber embedded with soot nanoparticles was stretched and released at least a thousand times to check its flexibility. Superhydrophobic soot coating was placed horizontally at a distance of 29 cm beneath a fully opened 0.5-m-diameter water tap in order to evaluate the plastron stability and its healing behavior. Due to high-pressure water, the plastron got damaged temporarily, and Cassie–Baxter state transit to the Wenzel state. The soot coating was exposed to ultraviolet light in order to ensure the healing of air plastrons on superhydrophobic soot coating under water.

### 2.9 Sailing boat test

The experiment was performed in a sink tank. Two boats were used, one with soot coating and one without coating. To direct the motion of the boat in a straight manner within the tank, 49.17 g of weight was tied along the string to each boat, as shown in Supplementary Figure S3. The distance covered by both boats was measured.

### 2.10 Load-bearing attribute

Two 99.9% pure aluminum plates (34.92 g each) with 8 cm × 8 cm dimensions were used to test the floating ability. One aluminum plate in its original state (uncoated) and the other coated with RTV-1 binder, followed by the soot coating (top, bottom, and edges), were submerged in water. The load-bearing capability of the prepared soot-coated aluminum plate was determined by placing 11 iron springy clips weighing 5.2 g (0.47 g mass of each iron clip) on its top face.

## 3 Results and discussion

### 3.1 Fabrication protocol of durable candle soot coating

**FIGURE 1:**
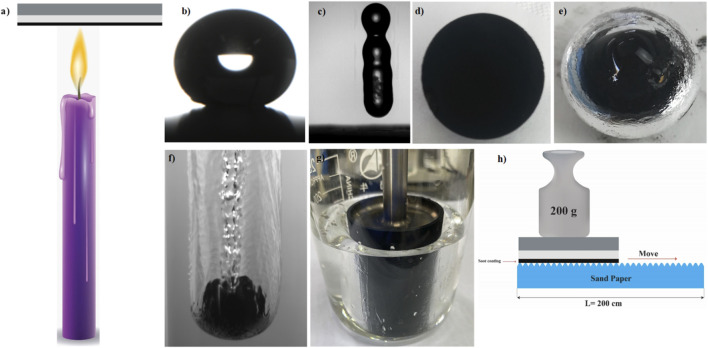
**(a)** Candle soot nanoparticles deposition into binder **(b)** Contact angle of water on soot coating **(c)** Jumping of water droplet on soot coating **(d)** Soot coated copper sphere **(e)** A shinning thin layer of trapped gas i.e. air plastron on soot coated sphere immersed in water **(f)** Cavity formation around soot coated sphere **(g)** Soot coated rotor immersed in water **(h)** Sand abrasion test on soot coating under load.

The bare candle soot nanoparticles are superhydrophobic but their weak bonding with the substrate results from unstable coating which cannot be used for practical applications. To address this issue, RTV is used as a binder to improve the surface adhesion property and durability of candle soot coating in harsh environmental conditions. RTV (room temperature vulcanizing) silicone rubber has hydrophobic, chemical resistant, and low modulus properties. RTV showed strong binding affinity not only with the substrate but also among candle soot nanoparticles. After soot deposition into the binder, a durable elastic superhydrophobic coating was obtained which showed the jumping phenomenon of water droplets due to the flexibility of the developed soot coating as shown in Figures 1a–c. The cured RTV retained its flexible, rubber-like thin film due to its heat-resistant and non-deformable characteristics Figures 1d–h.

**FIGURE 2:**
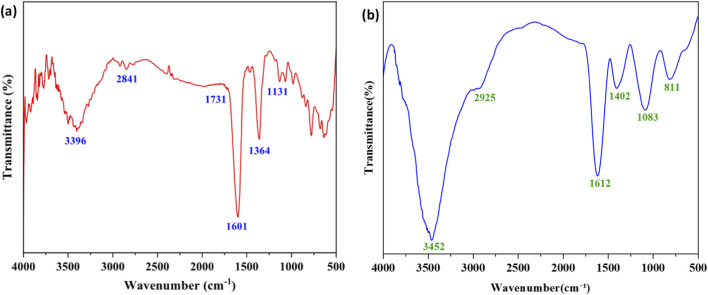
**(a)** Fourier-transform infrared spectra of candle soot particles **(b)** FTIR analysis of RTV-1 silicon rubber.

FTIR (Fourier-transform infrared) results show that the candle soot particles have broad band of O-H stretching at 3,396 cm^-1^ due to the presence of phenol, C-H stretching at 2,841 cm^-1^ due to the presence of aliphatic CH_2_-/CH_3_- from unburnt paraffinic wax and strong band at 1,601 cm^-1^ due to the presence of C=C stretching of aromatic rings and Graphitic carbon. FTIR spectrum is shown in Figure 2a. Thus the candle soot particles are made of Graphitic carbon (elemental carbon) including polycyclic aromatic hydrocarbons.

The FTIR analysis of RTV-1 silicone revealed distinct absorption bands characteristic of a polysiloxane network. The broad peak at 3,452 cm^-1^ corresponds to O–H stretching vibrations of silanol groups, while the band at 2,925 cm^-1^ is attributed to asymmetric C–H stretching of methyl substituents bound to silicon as shown in Figure 2b. The peak at 1,612 cm^-1^ may be assigned to the vinyl group. A strong absorption at 1,402 cm^-1^ indicates Si–CH_3_ bending, whereas the intense band at 1,083 cm^-1^ is assigned to the Si–O–Si asymmetric stretching vibration, which is the defining feature of the siloxane backbone. The absorption at 811 cm^-1^ originates from Si–C stretching and Si–CH_3_ rocking vibrations. The adhesion between the soot particles and the RTV-1 is very strong due to the covalent bonding between the hydroxyl group (O-H) present on soot particles and the hydroxyl group of silanol.

### 3.2 BET measurements

**FIGURE 3:**
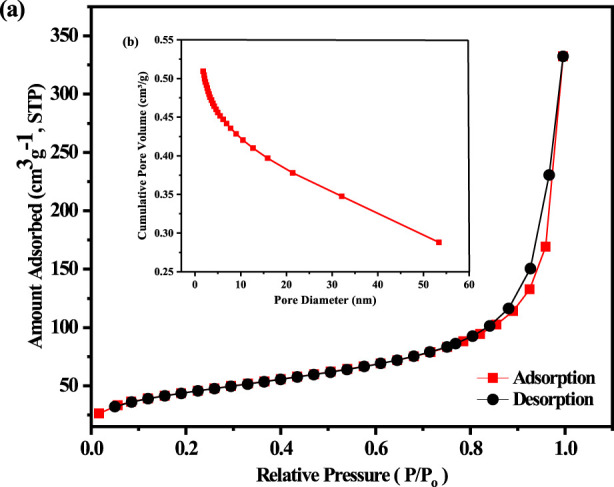
**(a)** BET $N_{2}$ sorption isotherm **(b)** BJH pore size distribution of the candle soot.

Using the BET method and the BJH desorption cumulative pore volume, the specific surface area of the sample was determined. The soot particles showed porous properties with surface area 156.6664 m^2^/g, average pore size 13.13247 nm, and average pore volume 0.514,354 cm^3^/g as shown in Figure 3. According to the Barret–Joyner–Halenda (BJH) pore size distribution curve confirmed that the surface area, average pore size, and average pore volume of the candle soot particles were 147.1270 m^2^/g, 13.7809 nm, and 0.506,885 cm^3^/g, respectively.

**FIGURE 4:**
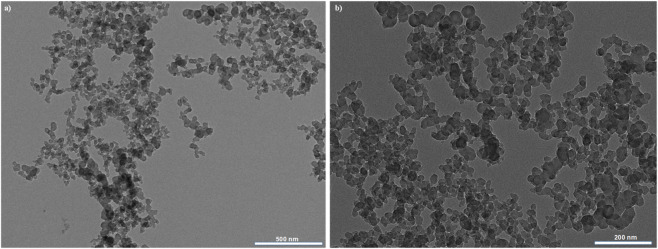
TEM images **(a,b)** of candle soot nanoparticles at different resolutions.

**FIGURE 5:**
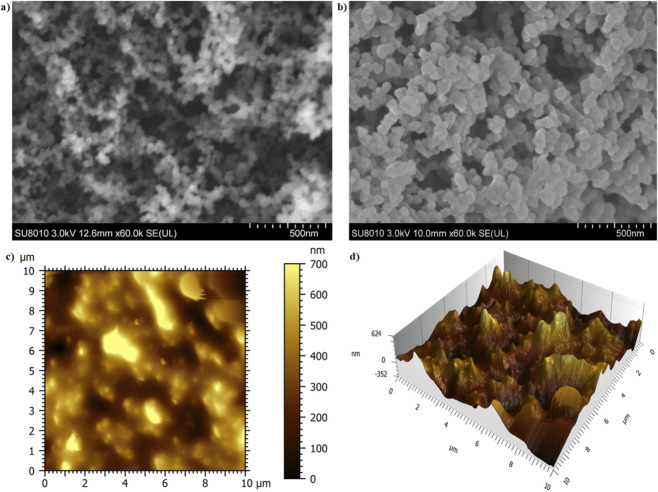
**(a)** Candle soot particles without binder. **(b)** Candle soot particles in the presence of binder. **(c)** 2-D AFM image of soot coating. **(d)** 3-D AFM image of soot coating.

In Figure 4, TEM images illustrate the fractal-like and aggregation behavior of the candle soot nanoparticles. It shows the highly ordered spherical, chain-like aggregates of carbon nanostructures. To achieve a small size of carbon nanoparticles and exceptional water repellency, the soot was collected from the upper part of the flame (2.9 cm above the candle wick. The SEM image of the candle soot nanoparticles with no binder demonstrates their porous morphology and an average diameter of 30 ± 7 nm as shown in Figure 5a. The SEM image in Figure 5b, displays the aggregation and embedding of candle soot nanoparticles within the binder. The topographical features of the surface coated with candle soot nanoparticles were analyzed using atomic force microscopy (AFM). The average root means square roughness (Rq rms) of the surface coated with soot nanoparticles was 106 ± 6 nm with R_A_ 87 ± 4 as shown in Figures 5c,d. By increasing the quantity of soot particles into the binder the developed coating showed more roughness as shown in the Supplementary Material S5.

**FIGURE 6:**
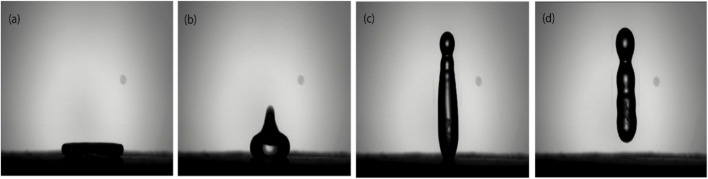
A water drop in contact with soot coating **(a)** pancake shape **(b)** singular jet **(c)** columnar jet **(d)** complete rebound without splashing.

In order to make the RTV binder coated substrate fully black, To and fro motion was applied for a fixed time of 2 min. The quantity of soot particles increases with the passage of time as shown in Supplementary Figure S2. The increase in amount of soot particles in the binder, the contact angle of water increases. The candle soot coating (CA 158° ± 4, water 10 µL) demonstrated superior water repellency in comparison to the simple RTV-1 (CA 100° ± 4, water 10 µL) and glass (CA 45° ± 4, water 10 µL) as shown in Supplementary Figure S4. The calculated water advancing and receding contact angles were 158° ± 2° and 159° ± 3° respectively. The superhydrophobic candle soot coating also demonstrated non-wetting behavior when a water droplet was impacted on it, as the kinetic energy was partially distributed into interfacial energy. When the water droplet just came into contact with the soot coating, it immediately spread to its maximum radius and then retracted to take on its original shape instantly because of the lower surface energy of the soot coating. The droplet was shaped like a columnar jet and bounced back fully without any sticking or splashing, as shown in Figure 6. As a result, the contact area of water on the superhydrophobic soot coating was reduced, and the contact angle increased.

### 3.3 Cassie–Baxter state of soot coating

When the soot coating was submerged in water, air was retained in the soot nanoparticles. The light is reflected from the layer of air trapped between the voids of the candle soot coating due to total internal reflection, it results in a silvery appearance shining like a mirror as shown in the Supplementary Video S1. This shiny look of the candle soot layer beneath water reflects its Cassie–Baxter superhydrophobic state, causing low contact angle hysteresis.

### 3.4 Sphere-in-cavity configuration

A significant perception in fluid mechanics regarding the motion of solid objects is the solid-liquid interface when submerged in water. Reducing the drag forces acting on these objects is a crucial consideration in this scenario to provide enhanced competency and substantial energy savings. A unique fascinating approach for reducing drag is the conception of near-zero drag, which involves enfolding firm spherical balls in water with gas cavities. By using a superhydrophobic soot coating on copper balls, the solid-gas-liquid interface was created, allowing free-slip boundary conditions instead of no-slip boundary circumstances. The spherical copper balls (diameters of 10 mm and 20 mm) were completely coated with candle soot particles to establish free-slip boundary conditions.

When these soot-coated copper balls (with mass m_S_ = 4.42 g and 35.14 g for 10 mm and 20 mm respectively) were submerged in water, the consequent formation of an air plastron around the entire coated sphere revealed the sphere-in-cavity, as shown in Supplementary Figure S6. Afterward, the sphere-in-cavity assembly was investigated for moving soot-coated copper balls when the balls were let to drop from a height of 90 cm in the air to a particularly designed, water-filled deep tank. A stable gas cavity with a teardrop form enfolded these copper balls when the balls moved with proper impact velocity. During the descendant, tiny gas bubbles were shed from the end extension of the gas cavity, enclosing the soot-coated copper balls, until they achieved steady falling velocity and reduced solid-liquid contact. The cavity’s capability to govern its teardrop shape allowed it to adjust its dimensions for the least drag, characteristically taking on a streamlined form to diminish resistance and realm its stability. The downfall of copper balls was recorded using high-speed and high-resolution video cameras, and the snapshots taken from video clips at high magnification were used to estimate cavity volume.

The volume of the copper ball and gas cavity surrounding the soot-coated copper ball was calculated by using the relations V_S_ = π/6 D_S_^3^ and V_C_ = 0.46LD^2^. Where D_S_ is the diameter of the sphere, L is the length of the cavity, and D is the maximum diameter of the cavity. For a soot-coated ball with a 10 mm diameter, the volume of the cavity (V_C_) is 9.26 times greater than the volume of the ball sphere (V_S_), where the length (L) and diameter (D) of the cavity are 61.40 mm and 13.09 mm, respectively. For a soot-coated ball with a diameter of 20 mm, V_C_ is 14.83 times greater than the V_S_, where the values of L and D, are 149 mm and 30.10 mm, respectively. An illustration of the sphere in cavity configuration for soot soot-coated copper ball is shown in Supplementary Figure S7 with certain parameters.

The extent of the drag coefficient (C_D_) was estimated by examining high-speed video footage of the gas cavity surrounding the copper sphere and using the relation relating the drag force with buoyancy forces and gravity on a falling sphere-in-cavity, as explained in the Supplementary Material. The near-zero drag coefficient (C_D_) ≈ −0.89 and −0.94 for the candle soot-coated copper spherical balls (D_S_ = 10 mm and 20 mm) respectively, confirmed the efficient drag reduction on superhydrophobic candle soot-coated copper balls as compared to uncoated balls. The near-zero drag coefficient, *C*_D_ ≈ 0.02 reported by Vakarelski, Ivan U., et al. for steel sphere-in-cavities signifies the cavity being neutrally buoyant. Here, for C_D_ ≈ −0.89 and −0.94, the negative values of the drag coefficient are attributed to buoyancy and high load-bearing properties. These properties are highly beneficial for vehicles moving in water with a high load and moving at speed with the lowest sinking probability.

**FIGURE 7:**
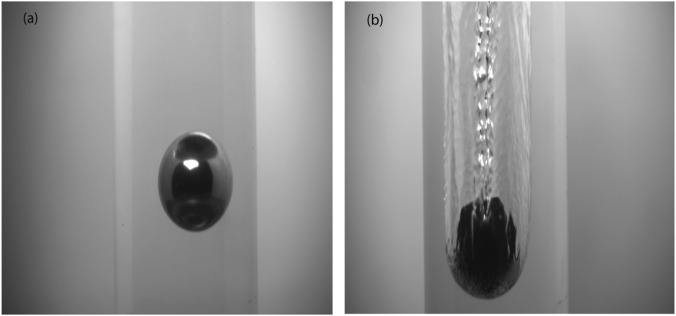
Creation of a gas cavity surrounding an impacting copper sphere with a 20-mm diameter. High-resolution snapshots from the movies; **(a)** Bare spherical copper balls **(b)** Formation of the gas cavity around soot-coated spherical copper.

So, due to the formation of air plastrons around the soot-coated copper balls and their encapsulation within the cavity, the drag force acting upon it was highly reduced. Due to the drag reduction, their speed was increased as compared to uncoated copper spherical balls within water. The larger the cavity formed, the higher the velocity. So, the soot-coated copper ball moved with high speed and covered more distance than the uncoated copper ball as shown in Figure 7. This novel way reduces drag and improves the system proficiency by utilizing ideas of fluid dynamics and surface science (Vakarelski et al., 2017).

### 3.5 Torque signal reduction on Taylor-Couette rotor

**FIGURE 8:**
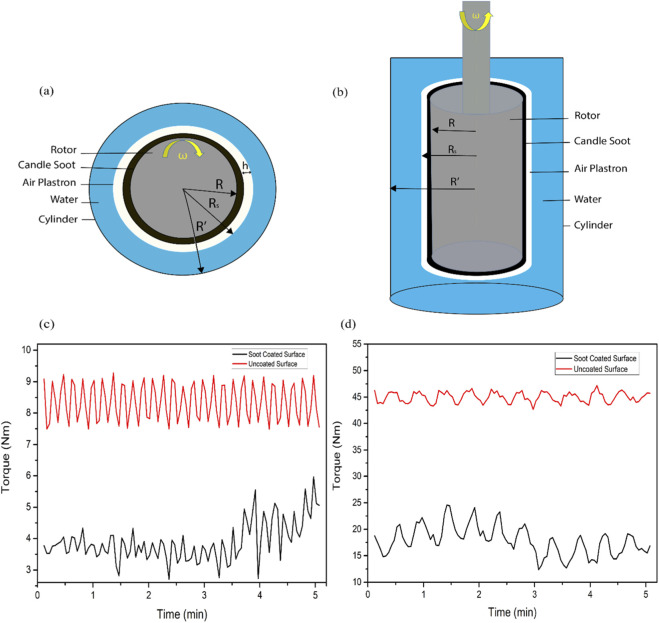
Schematic representations of the Taylor-Couette cell setup **(a)** The inner rotor, with a radius of (Ri) rotating at room temperature, produces an air plastron with a thickness (h) within a radius of (Ro), **(b)** Front view of the entire assembly with the soot. Torque measurement as a function of time at angular velocity at **(c)** w = 60 rad/s, **(d)** w = 240 rad/s.

The soot-coated Couette rotor and uncoated rotor were immersed in water at room temperature. The soot-coated rotor exhibited a thin continuous air plastron, resulting in a distinctive silvery appearance. The appearance of a silvery color indicates the Cassie–Baxter state of the coating. While no silvery color appears in the case of an uncoated rotor. The schematic representation of the Taylor-Couette cell setup is shown in Figures 8a,b. The Taylor-Couette rotor was rotated at a fixed angular velocity of 60 rad/s, the value of torque for the uncoated rotor was observed at 8.356 ± 0.601 Nm while the torque value for soot coated rotor was observed at 3.888 ± 0.629 Nm as shown in Figure 8c. The thin colored air layer formed on soot coated rotor within water is responsible for reducing additional torque under rotation. Likewise, when the bare rotor was rotated at an angular velocity of 240 rad/s, the observed value of the torque on the uncoated rotor was 44.976 ± 0.982 Nm while the soot-coated rotor showed a torque value at 17.861 ± 2.714 Nm; as shown in Figure 8d. Thus 60% reduction in torque was noted on soot soot-coated rotor as compared to the uncoated rotor.

### 3.6 Rheological experiments

**FIGURE 9:**
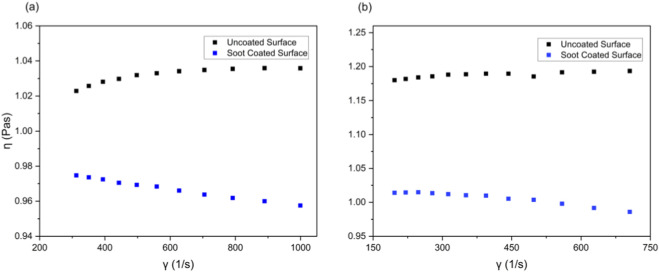
The apparent viscosity as a function of the shear rate at a gap height of 500 µm **(a)**, and 1000 µm **(b)**.

Regardless of the shear rate, the viscosity exhibited by the Newtonian fluid always remains constant. The viscosity of a Newtonian fluid glycerol was investigated for soot-coated and bare surfaces at different gap heights (h = 500 μm and 1,000 µm). The apparent viscosity was observed as a function of the shear rate and the mean apparent viscosity was computed at each gap height over the total series of investigated shear rates. The value of apparent viscosity as a function of the shear rate for uncoated surface and soot-coated surface at gap height (h = 500 µm) was calculated as 1.105 ± 0.005 Pas and 0.997 ± 0.005 Pas respectively. The reduction in apparent viscosity for the soot surface coated surface was 12.49% at the same gap height as compared to the bare surface as depicted in Figure 9a. At gap height (h = 1000 µm), the value of apparent viscosity for the uncoated surface and soot-coated surface was 1.187 ± 0.004 Pas and 1.006 ± 0.009 Pas respectively, as shown in Figure 9b. The 15.26% reduction in apparent viscosity on soot coated surface at the same gap height validated the drag reduction due to partial fluid slip (Ming et al., 2011; Srinivasan et al., 2013).

### 3.7 Durability and stability of air plastron

The presence of different ions in water, extreme pH and temperature conditions, and diffused gases can all harm the stability of the air plastron (Tesler et al., 2024). Plastron’s instability leads to poor durability of superhydrophobic coatings, which is a significant barrier for their large scale use in real marine environment. Commonly, the natural aquatic environment, cold conditions, acidic and basic media, saline conditions as well and organic solvents damage the superhydrophobic properties by deteriorating their surface topography. When plastrons get damaged, the solid-liquid interaction increases, which over time causes corrosion or biofouling, and eventual total material damage underwater. The formation of a continuous stable air plastron layer on soot coating and its outstanding chemical resilience is examined in super-cold water (−2 °C), liquid nitrogen (−180 °C), natural lake water, pond water, acidic/basic medium, and artificial seawater tests.

To determine the stability of the superhydrophobic candle soot coating, a succession of experiments was conducted. The soot coating was immersed for 6 months in deionized water with a pH of 7 at room temperature. It was observed that the Cassie–Baxter superhydrophobic state persistently existed. Because the light was reflected from the air plastron formed between the soot particle and the water layer, the dipped coating gave an appearance just like a shiny mirror in the water. Subsequently, when water droplets impacted a dry soot coating, they slid over like beads.

The stability of the soot coating at low temperatures was determined by immersing it in super-cold water (−2 °C). The coating was indeed able to form a continuous stable plastron layer even in extremely cold water. The Cassie–Baxter state was observed as the coating gave the appearance of a shiny mirror in water. Consequently, the super cold water was poured on the soot coating, and it remained stable, also the water droplets beaded up and rolled off the coating. This demonstrates that soot coating has a high degree of stability even at low temperatures, which makes it suitable for marine systems with near-zero temperatures. The results are shown in the Supplementary Video S2.

**FIGURE 10:**
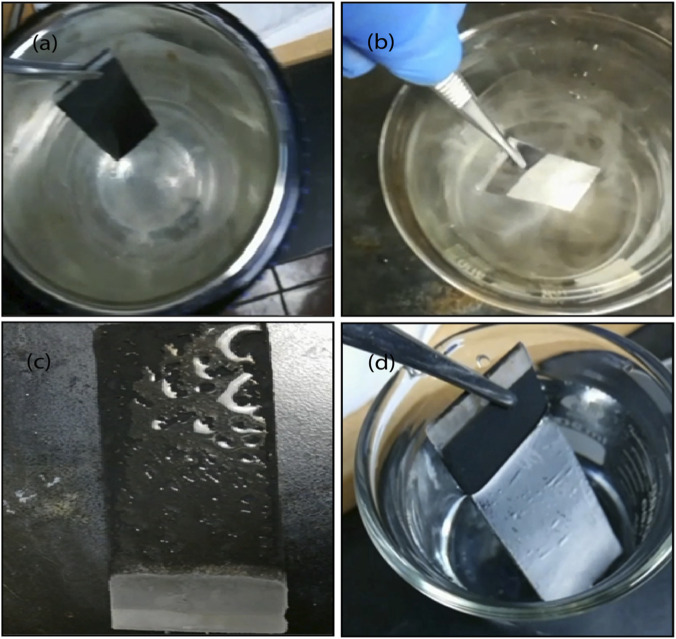
Nitrogen/water cycles. Candle soot coating **(a)** dipped in liquid nitrogen (−180 °C temperature), **(b)** dipped in water, **(c)** coating covered with frost layer, **(d)** Cassie–Baxter state.

To provide further evidence of the ability of prepared soot coating to regenerate stable plastron was tested by subjecting it to even extremely low temperatures (Esmeryan et al., 2022), in liquid nitrogen (≈−180 °C temperature). The coating was initially immersed in liquid nitrogen and then immediately dipped into water as shown in Figure 10a and (b) respectively. When the coating was removed from the water, it got a frost layer on it, Figure 10c. Once the frost melted away, the soot coating was dipped into water to perceive the Cassie–Baxter state. The coating was still able to regenerate a stable air plastron layer. Light reflected off from the air plastron formed between the dipped coating and the water, just creating a shiny mirror in the water as shown in Figure 10d and in the Supplementary Video S3. Subsequently, when the water droplets came into contact with the coating, they rolled off like beads from the coating. This cycle was performed 10 times and prepared superhydrophobic candle soot coating was even able to regenerate air plastrons consistently and highly maintained its superhydrophobicity.

Biofouling is a global issue for underwater vehicles that costs billions of dollars annually. Superhydrophobic coatings are crucial in preventing biofouling, but they are still somewhat unreliable, especially because of the poor stability of plastron underwater (Tesler et al., 2024). So here, presenting a solution to this problem, the superhydrophobic soot coating showed an outstanding anti-biofouling property by the creation of continuous stable air plastron. The coating was even able to retain its stability after 8 months of prolonged immersion in lake and pond water separately. Afterward, when water droplets were impacted on it, they rolled off like beads, showing the outstanding stability of the candle soot coating. Immersion of candle soot coating in lake and pond water is shown in Supplementary Material in Supplementary Figures S8, S9 respectively.

A step further, to assure the chemical stability of superhydrophobic coating, the pH impact of basic and acidic solutions was investigated it. Subsequently, the surface was immersed in basic and acidic mediums within a sealed experimental container to check its durability.

The soot coating’s resistance towards basic alkaline conditions was validated by exposing it to a 1 M NaOH solution (pH 10) for 7 days within a sealed experimental container, as shown in Supplementary Figure S10(a). The coating maintained its stability even after immersion, drying, and subsequent neutralization with pure water and CA remained above 150° with contact angle hysteresis consistently below 10°. To test its resistance towards acid, the coating was dipped for 7 days in a 1 M H_2_SO_4_ solution (pH 1.7), as shown in Supplementary Figure S10 (b) in the Supplementary Material. Pre- and post-immersion contact angles were computed, and over this period the water-repellent soot coating maintained passive air plastron stability constantly, as shown by the high contact angle and low contact angle hysteresis. Likewise, superhydrophobic soot coating was immersed in artificial seawater for 4 months, as shown in Supplementary Figure S11. The superhydrophobicity of the coating was restored when it was removed from seawater, as water droplets rolled off when they contacted the dried soot coating. When the coating was dipped in water, it gave a shiny appearance as the coating was quite able to regenerate the air plastron layer showing the Cassie–Baxter state.

Various organic solvents can react with the superhydrophobic coatings when they come in contact for long durations, thus damaging the air plastrons. So, to assess the plastron stability of candle soot coating, it was exposed to several solvents (mentioned above in Section 2.7). After the evaporation of solvents from the soot coating. When the dried soot coating was dipped in water, it efficiently regenerated the air plastron layer giving a shining mirror appearance confirming the Cassie–Baxter state. This study confirms the continuous stable air plastrons of the soot coating, demonstrating its extraordinary resistance to super-cold water (−2 °C), liquid nitrogen (−180 °C), natural lake and pond water, acidic-basic media, artificial seawater, and organic solvents making it highly valuable for marine engineering applications. Milk, coffee, honey, ethylene glycol, glycerol, and propylene glycol roll off easily and do not contaminate the soot coating as shown in Supplementary Figure S12.

Superhydrophobic soot coating also exhibits self-cleaning properties (Sutar et al., 2021) due to the presence of air plastron and Cassie–Baxter state underwater. To demonstrate the self-cleaning capability of prepared coating, dust was purposely spotted onto the soot coating that was positioned at a slope angle of ∼10° and subsequently rinsed with water. A digital camera was used to capture the self-cleaning consequence of coating. Few water droplets were introduced to the soot-coating surface, and the water droplets instantly rolled off, taking dust particles along with them. A spotless, clean, and clear surface was achieved as the dust simply gets rolled off with water droplets as shown in Supplementary Video S4, and Supplementary Figure S13. Nevertheless, the uncoated surface remains uncleaned even after rinsing with plenty of water. This suggests that the prepared coating has a lotus leaf like self-cleaning characteristic, because of which the superhydrophobic coating can shield the substrates from contaminants, so will remain advantageous for further use.

### 3.8 Mechanical durability

Using the sand abrasion test, the created coating’s mechanical durability was evaluated. First the developed soot coating was abraded by attaching small piece of sand paper beneath the 200 g weight then, 200 g weight was placed on the prepared soot coating and it was abraded on 400-grid silicon carbide sandpaper. To abrade the coating, it was propelled in one direction, up to a distance of 200 cm (20 cm per cycle). After repeating this abrasion test for 10 cycles, the coating was immersed in water to determine its Cassie–Baxter superhydrophobicity. Light reflection from air plastrons trapped between soot particles and the water layer caused the dipped coating to seem like a shiny mirror in water. Sand abrasion test and formation of stable air plastron layer are shown in Supplementary Figure S14 and Supplementary Video S5.

**FIGURE 11:**
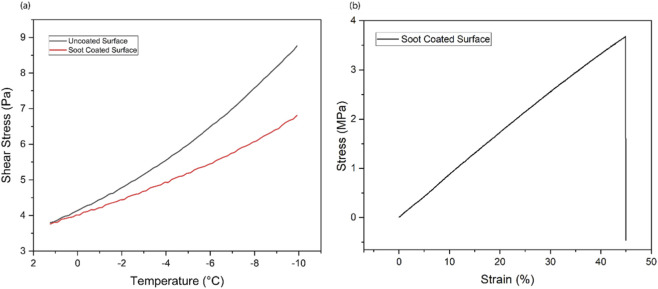
**(a)** Shear stress as a function of temperature for candle soot coating. **(b)** Shear stress-strain curve of candle soot-coated surface.

A step further, to ensure the mechanical stability of superhydrophobic coating, the shear-stress test was performed at low temperatures (1 °C to −10 °C) as shown in Figure 11a. For the bare surface, the shear rate was computed at 3.87 Pa, at 1 °C temperature, which raised up to 8.75 Pa (5.970 ± 1.441 Pa) at a low temperature of −10 °C. Meanwhile, at the same range of low temperature, shear stress for soot coated surface only raised from 3.81 Pa to 6.80 Pa (5.143 ± 0.878 Pa) indicated by the red line. Reduction in shear stress as a function of temperature was 13.86%, revealing the stability of soot coating and better mechanical strength at low temperatures (Yao et al., 2015). The cured silicon rubber embedded with soot particles was stretched and released at least 100 times and it returned to its original shape without cracking, which confirms its elasticity and flexibility. The stress-strain test was performed for soot coating with dimensions of 15 mm × 2.5 mm × 0.6 mm. The coating was clamped by the clips and subjected to over 100 cycles of stretching and releasing using an electric tensile rig providing maximum force up to 50 N. The coating was able to withstand a load of 2.5 N. A good mechanical strength was demonstrated by the prepared soot coating with 3.68 MPa tensile strength and 44.91% elongation, as shown in Figure 11 (b) (Zhang et al., 2013).

### 3.9 Healable air plastrons

**FIGURE 12:**
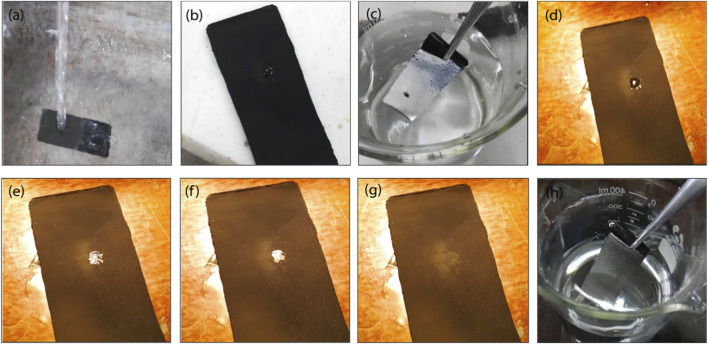
Candle-soot based superhydrophobic coating with healable plastron; **(a)** coating placed horizontally under water tap, **(b)** transition from Cassie–Baxter state to the Wenzel state, **(c)** Wenzel state (black spot) visible, **(d–g)** soot coating placed under UV lamp where Wenzel state transits to Cassie–Baxter state showing healable plastron, **(h)** Cassie–Baxter state confirmed by sheen when coating dipped in water.

Researchers have been indulged for years in making such superhydrophobic coatings that can withstand plastron damage and self-heal to retain their superhydrophobicity. By using various expensive techniques plastron healing is somehow possible, but a superhydrophobic coating having the capability of self-healing plastron has always been a great unsolved challenge. Here the candle soot superhydrophobic coating showed healable plastrons as soon as the water/solvent evaporated from the coating. For this, superhydrophobic soot coating was placed horizontally at a distance of 29 cm beneath a 0.5-m-diameter water tap. Then water was dripped on the soot coating drop by drop for a few minutes. After that, the flow of water was progressively increased until the tap was fully opened and high-pressure water streamed on the coating, leading it to change from the Cassie–Baxter state to the Wenzel state, as shown in Figures 12a,b. The coating displayed a shiny appearance when submerged in water, except for a single black spot observed in the coating under water indicates the Wenzel state, Figure 12c. The coating transits from the Wenzel to the Cassie-Baster state just when exposed to UV light for one and half minutes only, Figures 12d–g. The Cassie-Baster state of the developed coating was confirmed by the generation of the plastron and the observation of the sheen upon dipping it in water as shown in Figure 12h and Supplementary Video S6.

### 3.10 Sailing boat speed

The floating ability of soot coating and buoyancy effect was confirmed by the comparative study of two tiny boats underwater; one with candle soot coating to make it superhydrophobic and the other one without coating. The superhydrophobic coating exhibits low surface energy and reduced water friction. This characteristic makes it easier for the objects to slide on the water’s surface, which lowers resistance and improves their capacity to float. The boat without superhydrophobic coating covered 93 cm distance only. While, the other boat with soot coating travelled a 137 cm distance with high velocity as shown in Supplementary Figure S15 and Supplementary Video S9. The increased speed of the soot-soot-coated boats is ascribed to the creation of an air plastron that reduces drag. This suggested that the boat that has been treated with superhydrophobic soot coating is more buoyant, experiences less drag, and can float above water effortlessly. Therefore, this coating is suitable for underwater vehicles since it reduces drag, which not only increases speed but also efficiently reduces fuel consumption (Dong et al., 2013).

### 3.11 Buoyancy and load-bearing attribute

**FIGURE 13:**
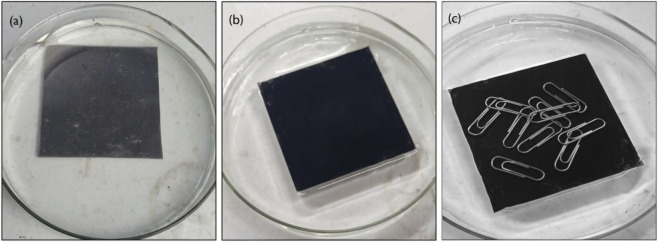
Aluminium plates: **(a)** without soot coating, submerged in water, **(b)** with soot coating, floated on the water surface, **(c)** floated on the water surface even after placing the load on its top surface.

The buoyancy enhancement driven by the superhydrophobic soot coating was determined by applying it on an 8 cm × 8 cm aluminium plate (99.9% pure). The density of the aluminium metal is 2.71 g/cm^3^, which is much greater than that of water (1.0 g/cm^3^). So, it effortlessly sinks into water and becomes readily wetting. Thus, as soon as an uncoated aluminium plate was put in water, it sank to the bottom as shown in Figure 13a. The weighed aluminium plate (34.92 g) was coated with RTV-1 binder (top, bottom, and edges), followed by the deposition of soot particles. When this soot deposited coating was placed on the surface of water, it was buoyant and floated on the water’s surface, and water dimples were observed around the soot-coated aluminium plate. An object floats on the water’s surface merely when its buoyant force is stronger than the force that would cause it to drown. Here, buoyancy is attributed to the thin air plastron created due to superhydrophobic soot coating, making it highly floatable as shown in Figure 13b.

The ability of a floating object to bear load is dependent on how it repels the water when it comes into contact with the water. The load-bearing capability of the soot-coated aluminum plate was determined by placing iron springs on its top surface during floating in water. 11 clips weighing 5.2 g (0.47 g mass of each) were loaded onto the plate surface; it did not soak, and it maintained its superhydrophobicity, demonstrating excellent floating and load bearing properties as shown in Figure 13c and Supplementary Video S7. Exceptional load-bearing and floating properties due to the superhydrophobic soot coating will remain advantageous for upcoming applications (Suyambulingam et al., 2017).

## 4 Conclusion

This work focuses on the synthesis of a durable superhydrophobic coating with a healing air plastron for drag reduction underwater, emphasizing its potential to maintain its durability in marine environments even at low temperatures. The candle soot nanoparticles are deposited into a viscoelastic RTV-1 binder to develop a robust, flexible, eco-friendly, and durable Cassie–Baxter superhydrophobic coating that effectively reduces drag underwater. The volume of the teardrop-shaped sphere-in-cavity surrounding the soot-coated copper ball is 14.83 times greater than the volume of the bare copper ball due to the passive air plastron stability when immersed in water. The developed coating is capable of reducing drag by 60% in Taylor-Couette flow. A 15.26% reduction in apparent viscosity validates drag reduction in rheological experiments. Shear stress reduction by 13.86% shows its low adhesion and enhanced mechanical strength at low temperatures. The developed soot coating is capable of retaining its superhydrophobicity due to the formation of a stable air plastron layer constantly in extremely cold conditions, natural aquatic mediums, highly acidic-basic media, artificial seawater, and even after treatment with various solvents. The sand abrasion test confirms the excellent mechanical properties exhibited by the coating. In particular, the superhydrophobic soot coating can heal the air plastron layer simply upon drying. The soot-coated boat covered more distance at high speed than the uncoated boat which confirms the drag reduction. Candle soot coating floats on the water’s surface even after being forcefully pushed into the water. Candle-soot superhydrophobic coating exhibits healable air plastrons with self-cleaning, anti-fouling, anti-corrosive, and load-bearing abilities. These properties of superhydrophobic candle soot coating make it a promising candidate for maritime transportation applications at subzero temperatures.

## Data Availability

The original contributions presented in the study are included in the article/Supplementary Material, further inquiries can be directed to the corresponding authors.

## References

[B1] Ahmadzadehtalatapeh M. Mousavi M. (2015). A review on the drag reduction methods of the ship hulls for improving the hydrodynamic performance. Int. J. Marit. Technol. 4, 51–64. Available online at: https://web.archive.org/web/20220801200145id_/http://ijmt.ir/files/site1/user_files_13d531/ahmadzadeh-A-10-541-1-1f68ae0.pdf.

[B2] Barbier C. Jenner E. D’Urso B. (2012). “Drag reduction with superhydrophobic riblets,” in ASME international mechanical engineering congress and exposition (American Society of Mechanical Engineers).

[B3] Bixler G. D. Bhushan B. (2012). Bioinspired rice leaf and butterfly wing surface structures combining shark skin and lotus effects. Soft Matter 8 (44), 11271–11284. 10.1039/c2sm26655e

[B4] Bixler G. D. Bhushan B. (2013). Fluid drag reduction with shark‐skin riblet inspired microstructured surfaces. Adv. Funct. Mater. 23 (36), 4507–4528. 10.1002/adfm.201203683

[B5] Brennan J. C. Geraldi N. R. Morris R. H. Fairhurst D. J. McHale G. Newton M. I. (2015). Flexible conformable hydrophobized surfaces for turbulent flow drag reduction. Sci. Reports 5 (1), 10267. 10.1038/srep10267 25975704 PMC4432562

[B6] Cassie A. Baxter S. (1944). Wettability of porous surfaces. Trans. Faraday Society 40, 546–551. 10.1039/tf9444000546

[B7] Chang A. Huang L. Wei S. Shao M. (2024). Analysis of the drag reduction performance and rheological properties of drag-reducing additives. Polymers 16 (9), 1247. 10.3390/polym16091247 38732715 PMC11085462

[B8] Chen Y. Hu Y. Zhang L.-W. (2024). Effective underwater drag reduction: a butterfly wing scale-inspired superhydrophobic surface. ACS Appl. Mater. and Interfaces 16 (20), 26954–26964. 10.1021/acsami.4c04272 38713183

[B9] Cheng M. Zhang S. Dong H. Han S. Wei H. Shi F. (2015). Improving the durability of a drag-reducing nanocoating by enhancing its mechanical stability. Acs Appl. Mater. and Interfaces 7 (7), 4275–4282. 10.1021/am5085012 25644454

[B10] Dong H. Cheng M. Zhang Y. Wei H. Shi F. (2013). Extraordinary drag-reducing effect of a superhydrophobic coating on a macroscopic model ship at high speed. J. Mater. Chem. A 1 (19), 5886–5891. 10.1039/c3ta10225d

[B11] Erbil H. Y. (2020). Practical applications of superhydrophobic materials and coatings: problems and perspectives. Langmuir 36 (10), 2493–2509. 10.1021/acs.langmuir.9b03908 32049544

[B12] Esmeryan K. D. Fedchenko Y. I. Gyoshev S. D. Lazarov Y. Chaushev T. A. Grakov T. (2022). On the development of ultradurable extremely water-repellent and oleophobic soot-based fabrics with direct relevance to sperm cryopreservation. ACS Appl. Bio Mater 5 (7), 3519–3529. 10.1021/acsabm.2c00457 35704856

[B13] Fakhri M. Rezaee B. Pakzad H. Moosavi A. (2023). Facile, scalable, and low-cost superhydrophobic coating for frictional drag reduction with anti-corrosion property. Tribol. Int. 178, 108091. 10.1016/j.triboint.2022.108091

[B14] Feng X. Sun P. Tian G. (2022). Recent developments of superhydrophobic surfaces (SHS) for underwater drag reduction opportunities and challenges. Adv. Mater. Interfaces 9 (2), 2101616. 10.1002/admi.202101616

[B15] Golovin K. B. Gose J. W. Perlin M. Ceccio S. L. Tuteja A. (2016). Bioinspired surfaces for turbulent drag reduction. Philosophical Trans. R. Soc. A Math. Phys. Eng. Sci. 374 (2073), 20160189. 10.1098/rsta.2016.0189 27354731 PMC4928507

[B16] Gu Y. Zhao G. Zheng J. Li Z. Liu W. Muhammad F. (2014). Experimental and numerical investigation on drag reduction of non-smooth bionic jet surface. Ocean. Eng. 81, 50–57. 10.1016/j.oceaneng.2014.02.015

[B17] Gu Y. Yu S. Mou J. Wu D. Zheng S. (2020). Research progress on the collaborative drag reduction effect of polymers and surfactants. Materials 13 (2), 444. 10.3390/ma13020444 31963432 PMC7013703

[B18] Hwang G. B. Patir A. Page K. Lu Y. Allan E. Parkin I. P. (2017). Buoyancy increase and drag-reduction through a simple superhydrophobic coating. Nanoscale 9 (22), 7588–7594. 10.1039/c7nr00950j 28537617

[B19] Jia H. Xie R. Zhou Y. (2022). Experimental investigation of the supercavitation and hydrodynamic characteristics of high-speed projectiles with hydrophobic and hydrophilic coatings. Fluids 7 (12), 363. 10.3390/fluids7120363

[B20] Kim M. Yoo S. Jeong H. E. Kwak M. K. (2022). Fabrication of Salvinia-inspired surfaces for hydrodynamic drag reduction by capillary-force-induced clustering. Nat. Commun. 13 (1), 5181. 10.1038/s41467-022-32919-4 36056031 PMC9440115

[B21] Liao K. Wang W. Mei X. Zhao W. Yuan H. Wang M. (2023). Stable and drag-reducing superhydrophobic silica glass microchannel prepared by femtosecond laser processing: design, fabrication, and properties. Mater. and Des. 225, 111501. 10.1016/j.matdes.2022.111501

[B22] Liravi M. Pakzad H. Moosavi A. Nouri-Borujerdi A. (2020). A comprehensive review on recent advances in superhydrophobic surfaces and their applications for drag reduction. Prog. Org. Coatings 140, 105537. 10.1016/j.porgcoat.2019.105537

[B23] Liu S. Sakai M. Liu B. Terashima C. Nakata K. Fujishima A. (2013). Facile synthesis of transparent superhydrophobic titania coating by using soot as a nanoimprint template. RSC Advances 3 (45), 22825–22829. 10.1039/c3ra43798a

[B24] Liu Y. Liu J. Tian Y. Zhang H. Wang R. Zhang B. (2019). Robust organic–inorganic composite films with multifunctional properties of superhydrophobicity, self-healing, and drag reduction. Industrial and Eng. Chem. Res. 58 (11), 4468–4478. 10.1021/acs.iecr.8b06302

[B25] Ming Z. Jian L. Chunxia W. Xiaokang Z. Lan C. (2011). Fluid drag reduction on superhydrophobic surfaces coated with carbon nanotube forests (CNTs). Soft Matter 7 (9), 4391–4396. 10.1039/c0sm01426e

[B26] Moaven K. Rad M. Taeibi-Rahni M. (2013). Experimental investigation of viscous drag reduction of superhydrophobic nano-coating in laminar and turbulent flows. Exp. Therm. Fluid Sci. 51, 239–243. 10.1016/j.expthermflusci.2013.08.003

[B27] Nilsson M. A. Daniello R. J. Rothstein J. P. (2010). A novel and inexpensive technique for creating superhydrophobic surfaces using teflon and sandpaper. J. Phys. D Appl. Phys. 43 (4), 045301. 10.1088/0022-3727/43/4/045301

[B28] Pakzad H. Liravi M. Moosavi A. Nouri-Borujerdi A. Najafkhani H. (2020). Fabrication of durable superhydrophobic surfaces using PDMS and beeswax for drag reduction of internal turbulent flow. Appl. Surf. Sci. 513, 145754. 10.1016/j.apsusc.2020.145754

[B29] Pakzad H. Nouri-Borujerdi A. Moosavi A. (2022). Drag reduction ability of slippery liquid-infused surfaces: a review. Prog. Org. Coatings 170, 106970. 10.1016/j.porgcoat.2022.106970

[B30] Papadopoulos P. Mammen L. Deng X. Vollmer D. Butt H. J. (2013). How superhydrophobicity breaks down. Proc. Natl. Acad. Sci. 110 (9), 3254–3258. 10.1073/pnas.1218673110 23382197 PMC3587223

[B31] Park H. Choi C.-H. Kim C.-J. (2021). Superhydrophobic drag reduction in turbulent flows: a critical review. Exp. Fluids 62 (11), 229. 10.1007/s00348-021-03322-4

[B32] Qu M. He J. Cao B. (2010). Facile fabrication of large-scale stable superhydrophobic surfaces with carbon sphere films by burning rapeseed oil. Appl. Surface Science 257 (1), 6–9. 10.1016/j.apsusc.2010.05.011

[B33] Su Y. Zhao Y. Jiang S. Hou X. Hong M. (2021). Anisotropic superhydrophobic properties of bioinspired surfaces by laser ablation of metal substrate inside water. Adv. Mater. Interfaces 8 (16), 2100555. 10.1002/admi.202100555

[B34] Saranadhi D. Chen D. Kleingartner J. A. Srinivasan S. Cohen R. E. McKinley G. H. (2016). Sustained drag reduction in a turbulent flow using a low-temperature Leidenfrost surface. Sci. Advances 2 (10), e1600686. 10.1126/sciadv.1600686 27757417 PMC5065253

[B35] Sarma B. Dalal A. Basu D. N. (2022). Interfacial dynamics of viscous droplets impacting a superhydrophobic candle soot surface: overview and comparison. Phys. Fluids 34 (1), 012121. 10.1063/5.0070828

[B36] Solomon B. R. Khalil K. S. Varanasi K. K. (2014). Drag reduction using lubricant-impregnated surfaces in viscous laminar flow. Langmuir 30 (36), 10970–10976. 10.1021/la5021143 25144426

[B37] Song D. Daniello R. J. Rothstein J. P. (2014). Drag reduction using superhydrophobic sanded teflon surfaces. Exp. Fluids 55, 1–8. 10.1007/s00348-014-1783-8

[B38] Srinivasan S. Choi W. Park K. C. Chhatre S. S. Cohen R. E. McKinley G. H. (2013). Drag reduction for viscous laminar flow on spray-coated non-wetting surfaces. Soft Matter 9 (24), 5691–5702. 10.1039/c3sm50445j

[B39] Sun P. Feng X. Tian G. Zhang X. Chu J. (2022). Ultrafast self-healing superhydrophobic surface for underwater drag reduction. Langmuir 38 (35), 10875–10885. 10.1021/acs.langmuir.2c01566 36001007

[B40] Sutar R. S. Latthe S. S. Sargar A. M. Patil C. E. Jadhav V. S. Patil A. N. (2020). Spray deposition of PDMS/candle soot NPs composite for self‐cleaning superhydrophobic coating. Macromol. Symp. 393 (1), 2000031. 10.1002/masy.202000031

[B41] Sutar R. S. Latthe S. S. Nagappan S. Ha C. Sadasivuni K. K. Liu S. (2021). Fabrication of robust self‐cleaning superhydrophobic coating by deposition of polymer layer on candle soot surface. J. Appl. Polym. Sci. 138 (9), 49943. 10.1002/app.49943

[B42] Suyambulingam G. R. T. Jeyasubramanian K. Mariappan V. K. Veluswamy P. Ikeda H. Krishnamoorthy K. (2017). Excellent floating and load bearing properties of superhydrophobic ZnO/copper stearate nanocoating. Chem. Eng. J. 320, 468–477. 10.1016/j.cej.2017.03.052

[B43] Tanaka T. Oishi Y. Park H. J. Tasaka Y. Murai Y. Kawakita C. (2023). Downstream persistence of frictional drag reduction with repetitive bubble injection. Ocean. Eng. 272, 113807. 10.1016/j.oceaneng.2023.113807

[B44] Tang Y. Cai Y. Wang L. Luo X. Wang B. Song Q. (2022). Formation mechanism of superhydrophobicity of stainless steel by laser-assisted decomposition of stearic acid and its corrosion resistance. Opt. and Laser Technol. 153, 108190. 10.1016/j.optlastec.2022.108190

[B45] Tesler A. B. Nurmi H. A. Kolle S. Prado L. H. Karunakaran B. Mazare A. (2024). Predicting plastron thermodynamic stability for underwater superhydrophobicity. Commun. Materials 5 (1), 112. 10.1038/s43246-024-00555-8

[B46] Vakarelski I. U. Patankar N. A. Marston J. O. Chan D. Y. C. Thoroddsen S. T. (2012). Stabilization of Leidenfrost vapour layer by textured superhydrophobic surfaces. Nature 489 (7415), 274–277. 10.1038/nature11418 22972299

[B47] Vakarelski I. U. Berry J. D. Chan D. Y. C. Thoroddsen S. T. (2016). Leidenfrost vapor layers reduce drag without the crisis in high viscosity liquids. Phys. Review Letters 117 (11), 114503. 10.1103/PhysRevLett.117.114503 27661694

[B48] Vakarelski I. U. Klaseboer E. Jetly A. Mansoor M. M. Aguirre-Pablo A. A. Chan D. Y. C. (2017). Self-determined shapes and velocities of giant near-zero drag gas cavities. Sci. Advances 3 (9), e1701558. 10.1126/sciadv.1701558 28913434 PMC5590785

[B49] Vega-Sánchez C. Peppou-Chapman S. Zhu L. Neto C. (2022). Nanobubbles explain the large slip observed on lubricant-infused surfaces. Nat. Communications 13 (1), 1–11. 10.1038/s41467-022-28016-1 35039515 PMC8764024

[B50] Wu X. Yang W. Liu Y. Wang X. Zhang Y. Ma S. (2024). Silicon gels with sustainable self-replenishment: fluid drag reduction through viscosity dependency. Tribol. Int. 194, 109460. 10.1016/j.triboint.2024.109460

[B51] Xu S. Lin J. Yu Y. Wang H. Lu J. (2023). Laminar drag reduction in a closed channel using bioinspired textured surfaces. Surf. Innov. 11 (6-7), 416–428. 10.1680/jsuin.22.01069

[B52] Yao X. Wu S. Chen L. Ju J. Gu Z. Liu M. (2015). Self‐replenishable anti‐waxing organogel materials. Angew. Chem. Int. Ed. 54 (31), 8975–8979. 10.1002/anie.201503031 26083324 PMC5033071

[B55] Yu C. Liu M. Zhang C. Yan H. Zhang M. Wu Q. (2020). Bio‐inspired drag reduction: from nature organisms to artificial functional surfaces. Giant 2, 100017. 10.1016/j.giant.2020.100017

[B53] Yunqing G. Tao L. Jiegang M. Zhengzan S. Peijian Z. (2017). Analysis of drag reduction methods and mechanisms of turbulent. Appl. Bionics Biomechanics 2017 (1), 6858720. 10.1155/2017/6858720 29104425 PMC5624150

[B54] Zhang W. Shi Z. Zhang F. Liu X. Jin J. Jiang L. (2013). Superhydrophobic and superoleophilic PVDF membranes for effective separation of water-in-oil emulsions with high flux. Adv. Mater. Deerf. Beach. Fla. 25 (14), 2071–2076. 10.1002/adma.201204520 23418068

[B56] Zhu S. Wu T. Bian Y. Chen C. Zhang Y. Li J. (2022). Sustaining robust cavities with slippery liquid–liquid interfaces. Adv. Sci. 9 (7), 2103568. 10.1002/advs.202103568 35037429 PMC8895157

[B57] Zulfiqar U. Hussain S. Z. Subhani T. Hussain I. (2018). Mechanically robust superhydrophobic coating from sawdust particles and carbon soot for oil/water separation. Colloids Surfaces A Physicochem. Eng. Aspects 539, 391–398. 10.1016/j.colsurfa.2017.12.047

