# Supplementary material for: Underwater drag reduction via self-healing and robust air plastrons stabilized by candle soot-based coatings
Source: Front Chem. 2026 May 1;14:1814633. doi: 10.3389/fchem.2026.1814633 (PMC13177182; doi:10.3389/fchem.2026.1814633)
Supplement: Supplementary file 7 [file DataSheet1.docx]

Supplementary Information

**Underwater Drag Reduction via Self-Healing and Robust Air Plastrons Stabilized by Candle Soot-Based Coatings**

Muhammad Imran Jamil^1^*, Arshia Komal^2^, Mehboob Hassan^2^, Mazloom Shah^1^, Waqar Ahmed^3^, Hafiz Muhammad Ali^4,5^, Numan Ahmed^6^, Fazal Haq^7^, Sahid Mehmood^8^, Shahid Iqbal^9^*, Meznah M. Alanazi^10^, Shaimaa A. M. Abdelmohsen^10^

^1^Department of Chemistry, Faculty of Science, Grand Asian University Sialkot, Punjab, Pakistan.

^2^Department of Chemistry, University of Narowal, Punjab, Pakistan.

^3^Department of Bionano Engineering, Hanyang University, Ansan 155-88, South Korea.

^4^Mechanical Engineering Department, King Fahd University of Petroleum and Minerals, Dhahran 31261, Saudi Arabia.

^5^Interdisciplinary Research Center for Sustainable Energy Systems (IRC-SES), King Fahd University of Petroleum and Minerals, Dhahran 31261, Saudi Arabia.

^6^Yangtze Delta Region Institute (Huzhou), University of Electronic Science and Technology of China, Huzhou, Zhejiang 313001, P. R. China

^7^Institute of Chemical Sciences, Gomal University, D.I.Khan, 29050, Pakistan.

^8^Key Laboratory for Special Functional Aggregated Materials of Ministry of Education, School of Chemistry and Chemical Engineering, Shandong University, Shandong, China.

*^9^Nottingham Ningbo China Beacons of Excellence Research and Innovation Institute, University of Nottingham Ningbo China, Ningbo 315100, China.*

*^10^Department of Physics, College of Science, Princess Nourah bint Abdulrahman University, P.O. Box 84428, Riyadh, 11671, Saudi Arabia.*

****To whom corresponding should be addressed***

imranjamil@gaus.edu.pk (MI Jamil) and [shahidgcs10@yahoo.com](mailto:shahidgcs10@yahoo.com), [shahidcbi8@outlook.com](mailto:shahidcbi8@outlook.com) (S. Iqbal)

**Figure S1.** Height and position of substrate in the candle flame.

**Figure S2.** Increasing quantity of candle soot nanoparticles with the passage of time.

**Figure S3**. a) Snapshot from the video. b) Two sailing boats, one without coating and other covered with soot coating, tied with 49.17 g weight through string.

**Figure S4.** Contact angle of water on various surfaces.

**Figure S5** a) 2-D AFM image of only RTV-1. b) 3-D AFM image of only RTV-1. c) 2-D AFM image of candle soot coating (CS+RTV-1) with more quantity of carbon soot. d) 3-D AFM image of candle soot coating (CS+RTV-1) with more quantity of carbon soot.

## Sphere-in-Cavity

For reducing drag, the creation of near-zero drag was achieved by enfolding the spherical cooper balls by gas cavities under water. By using a superhydrophobic soot coating on copper balls, the solid-gas-liquid interface was created, allowing the free slip boundary instead of the no slip boundary conditions. The spherical copper balls with diameter of 10 mm and 20 mm were taken. For each size, one ball was completely coated with a superhydrophobic candle soot coating and the other was used in an original, uncoated state during the experiment. When dipped in water, the soot-coated copper ball formed an air (gas) plastron around the entire area, revealing the sphere-in-cavity formation, as shown in Figure S5.


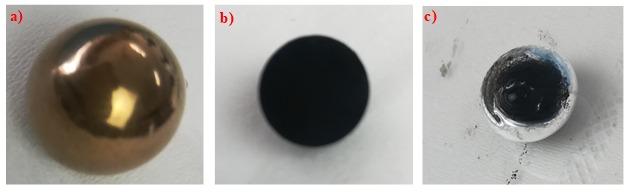


**Figure S6.** a) Original copper ball (uncoated), b) Soot coated copper ball, c) Plastron formed around soot coated copper ball within water.

To investigated the sphere-in-cavity assembly providing free-slip boundary conditions under water, the soot-coated copper balls (mass of sphere, m_S_ = 4.42 g and 35.14 g for diameter, D_S_ = 10 mm and 20 mm, respectively) were let to drop from a height of 90 cm in the air to a particularly designed, water-filled deep tank. A stable gas cavity with a teardrop form enfolded these copper balls when the balls moved with proper impact velocity. During descendant, tiny gas bubbles were shed from the end extension of the gas cavity, enclosing the soot-coated copper balls, until they achieved steady falling velocity and reduced solid-liquid contact. The cavity's capability to govern its teardrop shape allows it to adjust its dimensions for least drag, characteristically taking on a streamlined form to diminish resistance and realm its stability. The descent of copper balls was recorded using high-speed and high-resolution video cameras, and snapshots taken from video clips at high magnification were used to estimate sphere-in-cavity volume. Relations V_S_ = π/6 D_S_^3^ and V_C_ = 0.46LD^2^, were used to calculate the volume of the copper ball and gas cavity surrounding the soot-coated copper ball was respectively. The V_C_ computed was 9.26 and 14.83 times greater than the V_S_ for coated balls with 10 mm and 20 mm diameter, respectively. Illustration of sphere in cavity around soot coated copper ball is shown in Figure S6 with certain parameters.


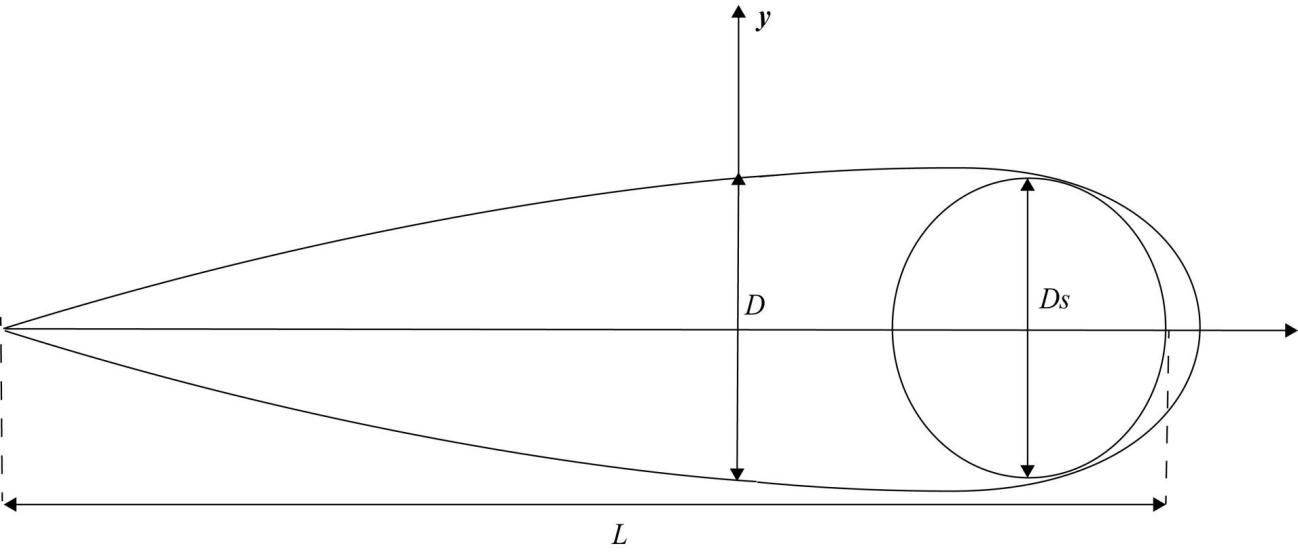


**Figure S7.** Illustration of sphere in cavity around for spherical soot coated copper ball when impacted from certain height in water.

The extent of the drag coefficient (C_D_) was also estimated by examining high-speed video footage of the gas cavity surrounding the copper sphere. To calculate C_D_, following relation was used to relate the drag force with buoyancy forces and gravity on a falling sphere-in-cavity at terminal velocity.

$$C_{D}=\frac{2g (m_{s}- \rho V_{C})}{\rho\pi R^{2}U^{2}}$$

Where, $g$ is gravity constant (9.8 m/s^2^), $\rho$ is density of water (997 Kg/m^3^) at 25 ℃, m_S_ is mass of copper sphere, $V_{C}$ is volume of cavity (including sphere), R is radius of copper sphere (D = 2R), $U$ is terminal velocity.

When the spherical copper ball with mass (m_S_ = 4.42 g) and radius (R = 5 mm) as d = 2R, was descended in 25 °C water with density ($\rho$= 997 Kg/m^3^) at terminal velocity ($U$ = 1.12 m/s). The volume of the cavity 4.849 mm^3^ (sphere-in-cavity including the sphere) surrounded the spherical ball. The near-zero drag coefficient (C_D_) ≈ *-*0.89 confirmed the efficient drag reduction on candle soot-coated copper spherical balls as compared to uncoated copper spherical balls. Likewise, when the copper ball with (D = 20 mm and m_S_ = 35.14 g) was descended in 25 °C water at $U$ = 2.02 m/s, the drag force activity on spherical ball was very low, as drag coefficient (C_D_) ≈ -0.94.

The near-zero drag coefficient (C_D_) ≈ -0.89 and -0.94 for candle soot-coated copper balls with diameter 10mm and 20 mm respectively, confirmed the proficient drag reduction on candle soot-coated copper spherical balls as compared to uncoated copper spherical balls. Due to drag reduction, its speed was also increased covering more distance as compared to uncoated copper spherical balls. The negative value of the drag coefficient owed to buoyancy and high load-bearing properties.^1^


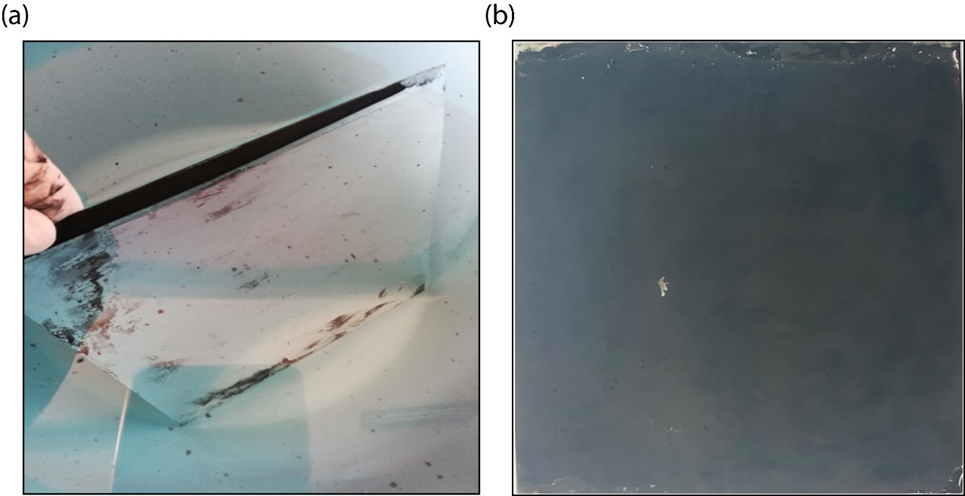


**Figure S8.** Candle soot coating, (a) immersed in lake water, (b) after 8-months immersion in lake water.


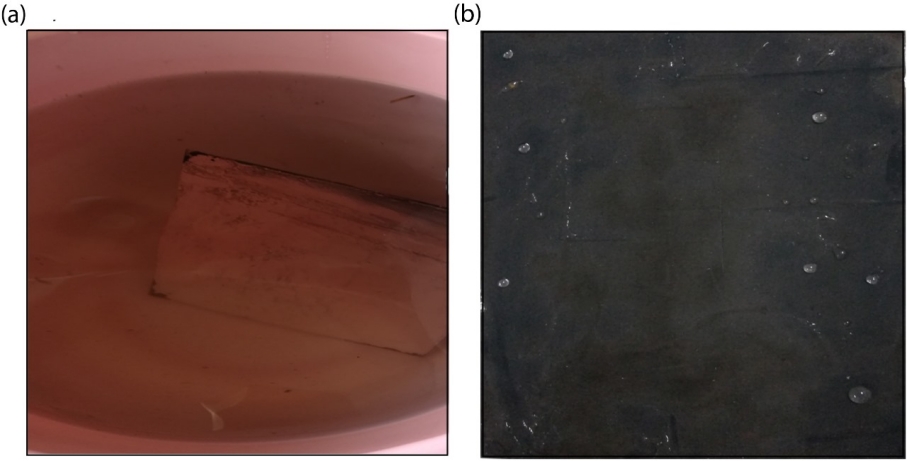


**Figure S9**. Candle soot coating, (a) immersed in pond water, (b) after 8-months immersion in pond water.


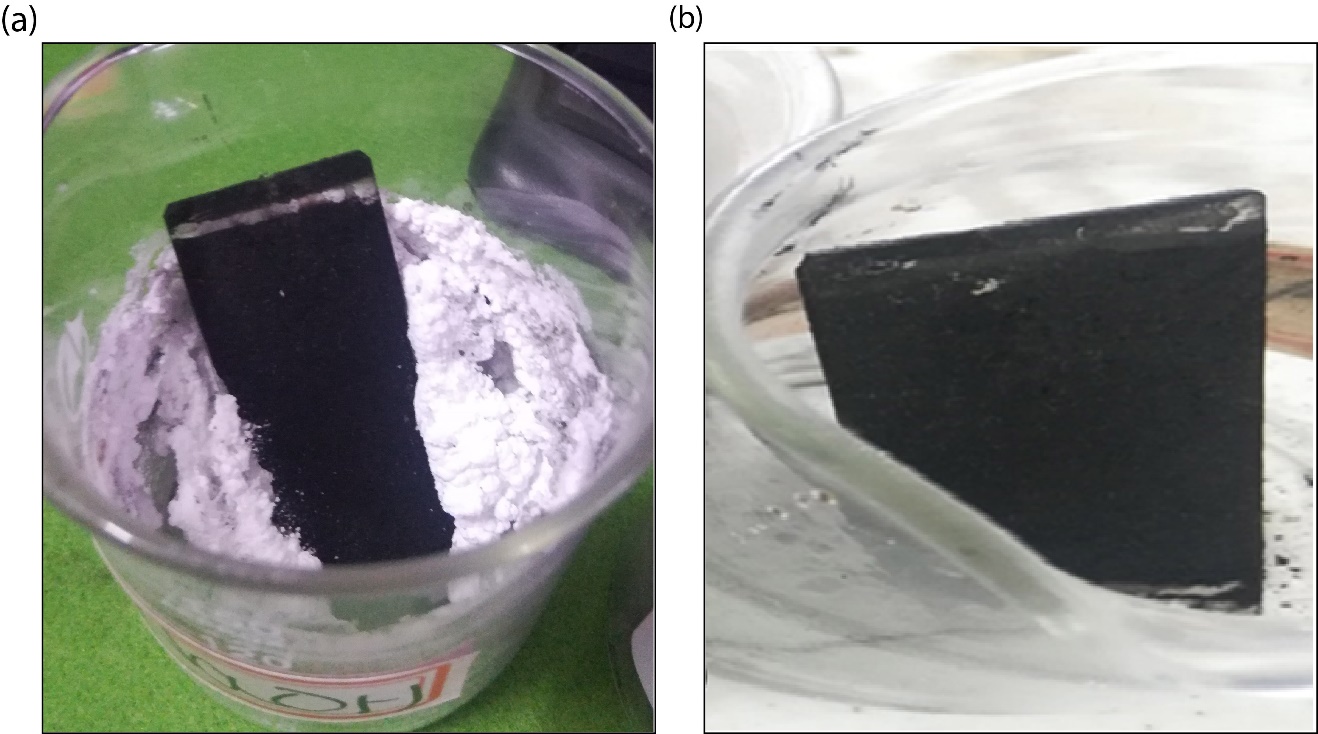


**Figure S10.** Candle soot coating immersed in a) basic medium, b) acidic medium.


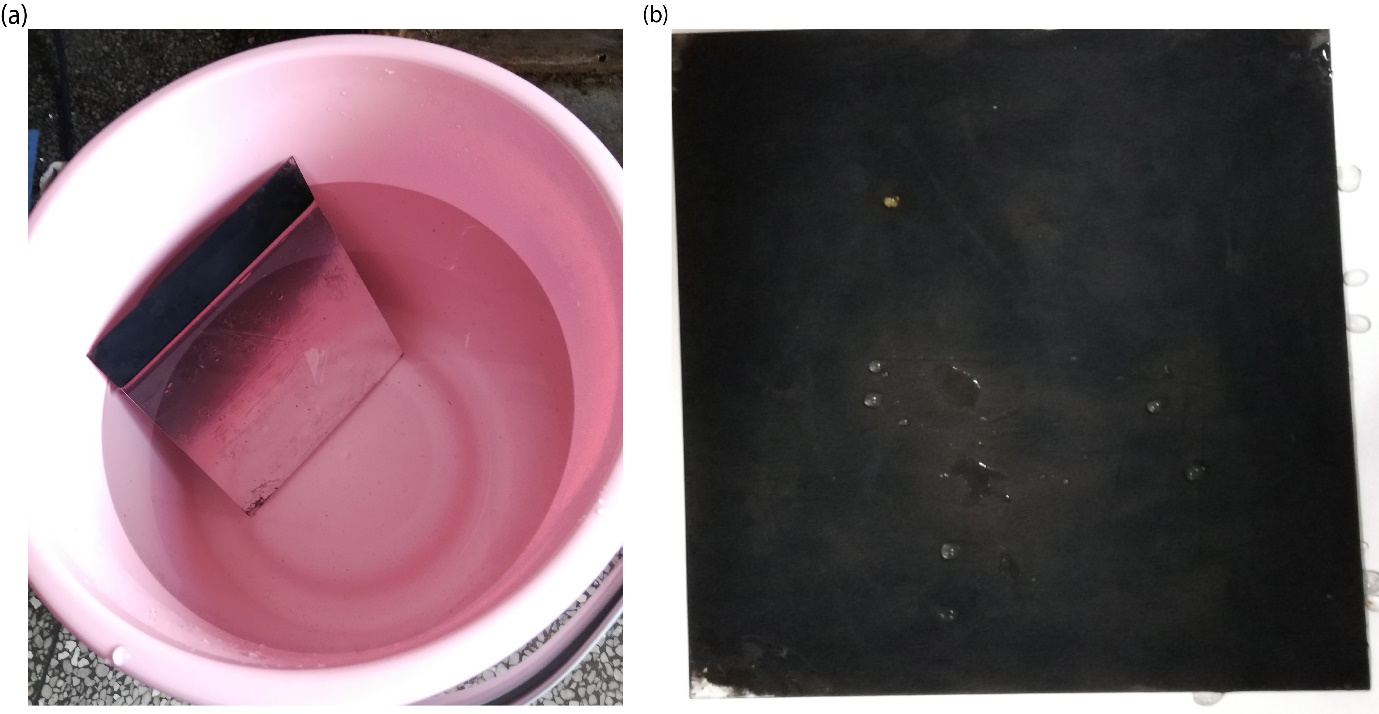


**Figure S11.** Candle soot coating, (a) immersed in artificial sea water, (b) after 4-month immersion in artificial sea water.

**Figure S12.** Repellence of milk (1), coffee (2), ethylene glycol (3), honey (4), propylene glycol (5), and glycerol (6) on candle soot coating.

**Figure S13.** Assessing self-cleaning property of candle soot coating; a) Spotting dust on coating angled at 10°. b) Water drops beading on the coating carrying the dust along it. c) Clean and clear candle soot coating.


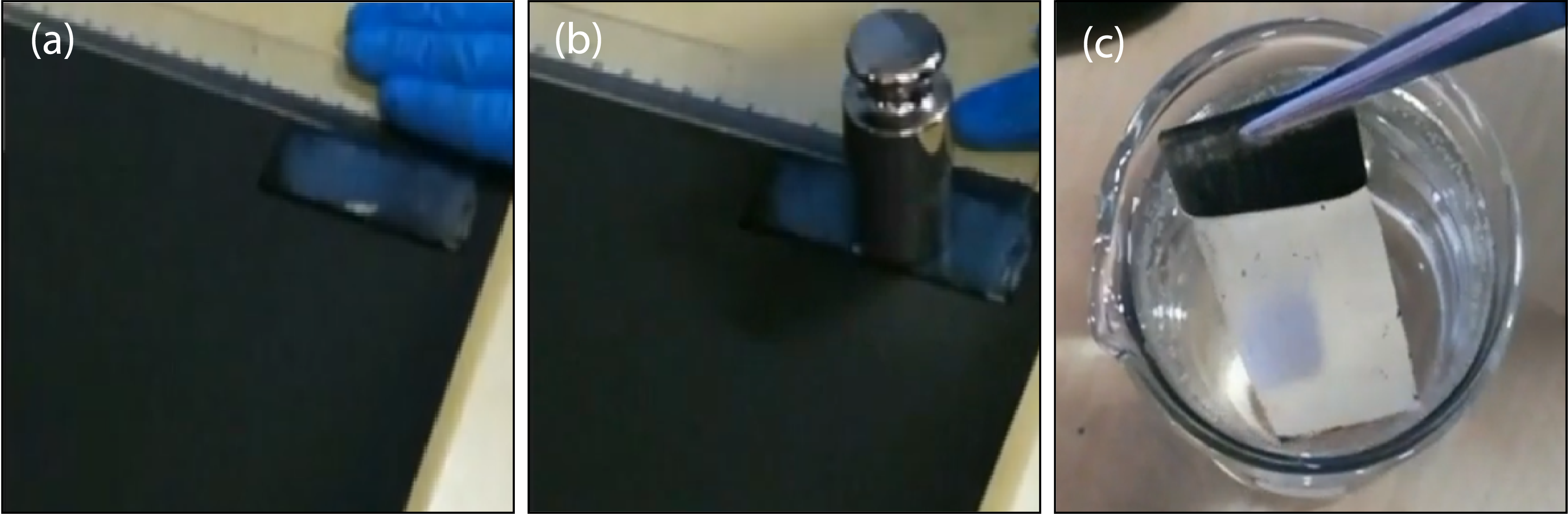


**Figure S14.** Sand abrasion test (a) candle soot coating placed on 400-grid silicon carbide sandpaper, (b) 200 g weight placed on soot coating, (c) After 10 cycles of abrasion, the coating exhibiting Cassie-Baxter state due to formation of stable air plastron layer.

**Figure S15.** a) Snapshot from the video. b) Assessment of sailing boat performance by applying soot coating, which covered more distance at high speed compared to the same boat without coating.

**Reference.**

1. Vakarelski, I. U.; Klaseboer, E.; Jetly, A.; Mansoor, M. M.; Aguirre-Pablo, A. A.; Chan, D. Y.; Thoroddsen, S. T., Self-determined shapes and velocities of giant near-zero drag gas cavities. *Science advances* **2017,** *3* (9), e1701558.
